# Type III Secretion System in Intestinal Pathogens and Metabolic Diseases

**DOI:** 10.1155/2024/4864639

**Published:** 2024-11-06

**Authors:** Le Zhou, Yaoyuan Zhang, Shiqi Wu, Yiyu Kuang, Pengfei Jiang, Xiao Zhu, Kai Yin

**Affiliations:** ^1^Guangxi Key Laboratory of Diabetic Systems Medicine, Guilin Medical University, Guilin 541100, China; ^2^Department of General Practice, The Fifth Affiliated Hospital of Southern Medical University, Guangzhou 510900, China

**Keywords:** diabetes, gut microbiota, intestinal pathogens, metabolic diseases, the type III secretion system

## Abstract

Modern lifestyle changes, especially the consumption of a diet high in salt, sugar, and fat, have contributed to the increasing incidence and prevalence of chronic metabolic diseases such as diabetes, obesity, and gout. Changing lifestyles continuously shape the gut microbiota which is closely related to the occurrence and development of metabolic diseases due to its specificity of composition and structural diversity. A large number of pathogenic bacteria such as *Yersinia*, *Salmonella*, *Shigella*, and pathogenic *E. coli* in the gut utilize the type III secretion system (T3SS) to help them resist host defenses and cause disease. Although the T3SS is critical for the virulence of many important human pathogens, its relationship with metabolic diseases remains unknown. This article reviews the structure and function of the T3SS, the disruption of intestinal barrier integrity by the T3SS, the changes in intestinal flora containing the T3SS in metabolic diseases, the possible mechanisms of the T3SS affecting metabolic diseases, and the application of the T3SS in the treatment of metabolic diseases. The aim is to provide insights into metabolic diseases targeting the T3SS, thereby serving as a valuable reference for future research on disease diagnosis, prevention, and treatment.

## 1. Introduction

Metabolic diseases encompass a cluster of disorders arising from aberrant metabolic processes. In light of the advancement and affluence of contemporary society, metabolic diseases have emerged as a prominent global public health concern [1]. The incidence and pervasiveness of metabolic diseases are intricately intertwined with lifestyle and dietary factors [2]. Nevertheless, the intricate interplay between these factors remains elusive.

Evidence is growing that the gut microbiota plays a significant role in various pathophysiological changes in the human body [3–5], such as defense against exogenous infection, immunological organs, and various aspects of metabolism. Gut microbiome dysbiosis primarily manifests due to changes in the composition of the microbiota, which may be caused by a variety of factors, including dietary changes, increased stress, abnormal levels of inflammatory markers, and antibiotic abuse. It often leads to the occurrence of metabolic diseases such as T2DM (type 2 diabetes mellitus) [6–9], gout [10–12], obesity [13, 14], and NAFLD (nonalcoholic fatty liver disease) [15].

By way of the long-term coevolution of pathogenic bacteria and human host cells, many Gram-negative bacteria, such as *Shigella*, *Yersinia*, enterohemorrhagic *Escherichia coli* (EHEC), enteropathogenic *E. coli* (EPEC), and *Salmonella* have evolved a complex type III secretion system (T3SS). The T3SS is crucial to bacterial pathogenicity. The effector proteins that enter human host cells via the T3SS can simulate and manipulate various signal transduction pathways in host cells through fine regulation, including apoptosis, autophagy, and the inflammatory response, thereby effectively escaping the defense response of host cells and enhancing infectivity and pathogenicity [16, 17].

It has been reported that the intestinal pathogenic flora attacks the mechanical barrier of the intestinal barrier via their T3SS [18–22]. The intestinal barrier creates a strong barrier that prevents toxic bacteria, viruses, and antigens from entering the intestinal environment, helping to absorb nutrients and ensuring normal immune function [23].

Although the intestinal flora plays an important role in the occurrence of metabolic diseases, the specific role of the T3SS in pathogenic bacteria is unknown. The effect of the T3SS in intestinal pathogens on metabolic diseases may be mainly achieved by affecting the function of the intestinal barrier.

This article reviews the relationship between the T3SS in the intestinal flora and metabolic diseases, providing a reference for the prevention and treatment of metabolic diseases.

## 2. A Short Review of the T3SS in Intestinal Pathogens

The T3SS is a pathogenicity island that encodes various virulence factors (VFs) present in bacterial genomes, and its size is approximately 30–40 kd. The T3SS in animal or plant pathogens is highly conserved in structure and consists of approximately 20 proteins [24, 25]. The structure of the pathogenic bacterial T3SS in different species is similar, and its structure resembles that of a syringe observed using transmission electron microscopy, so it is also called a needle-like complex [26].

As a component of pathogenic VFs, including extracellular polysaccharides, lipopolysaccharides (LPSs), extracellular enzymes, and type III effector proteins, the T3SS is widely distributed in Gram-negative bacteria and plays a decisive role in the process of bacterial infection in the host and pathogenesis [27]. In addition, T3SS effector proteins play an integral role in bacterial evasion of immune system attack. Early studies showed that knockout of the T3SS components significantly reduced the ability of bacteria to infect the host, or even led to its loss, but the survival of the bacteria was not affected [28].

The pathogenic bacteria within the gut with the T3SS include *Shigella* with the Mxi-spa system [29], *Yersini*a with the YOP system [30], *E. coli* with the locus of enterocyte effacement (LEE) and non-LEE-encoded VFs [31], and *Salmonella* with the SPI-1 and SPI-2 systems [32] (Table 1). These bacteria all belong to the Enterobacteriaceae family of the Gammaproteobacteria class, which can “inject” effector proteins into host cells via the T3SS to simulate and create a favorable environment for the bacteria by interfering with several signaling pathways in the host cells and improve bacterial survival so the bacteria can replicate and continue to spread, thereby enhancing their pathogenicity in the host cells [38, 39].

Under normal circumstances, the T3SS genes are not expressed. Appropriate environmental factors can activate the expression of the T3SS-encoding genes, thereby initiating the assembly of the needle-like complex bound to the double-cell membrane and the transport of various effector proteins [40–42]. The expression and secretion of T3SS effector proteins in the human intestine are activated by some chemicals, host exposure, growth conditions, and complex feedback control mechanisms. For example, *Yersinia* induces the expression of the T3SS gene *Yop* after contact with target cells, accompanied by the transfer of the T3SS effector protein YopE to the target cells [43–45].

## 3. The T3SS Disrupts Intestinal Barrier Integrity

The effect of the T3SS in intestinal pathogens on metabolic diseases is mainly achieved by affecting the function of the intestinal barrier.

On the one hand, the intestinal pathogenic flora destroys the tight junctions between intestinal epithelial cells via the effector proteins secreted. For example, *E. coli* can secrete NleA through the T3SS, destroying tight junction proteins [46]. *Shigella flexneri* can also interfere with the expression of the tight junction proteins ZO-1 and occludin through the T3SS [47]. The T3SS effector sopB in *Salmonella typhimurium* induces the expression of Snail, which regulates the genes of epithelial junction proteins and reduces the expression of three claudin proteins (ZO-1, claudin-1, and occludin) in the apical connexin [48–50].

On the other hand, some effector proteins of the T3SS in pathogenic bacteria can act on specific signal transduction pathways to induce the programmed death of intestinal epithelial cells to expand infection. During EPEC/EHEC infection, T3SS effector proteins, including EspF, Cif, and EspB, activate endogenous apoptosis in intestinal epithelial cells. For example, when EPEC infects cells, EspF targets the mitochondria, causing the loss of the mitochondrial outer membrane potential, the release of cytochrome C, and the cleavage of caspase-9 and caspase-3 [51]. Another study showed that EspF binds to Abcf2 of the ABC transporter family, and during infection, Abcf2 is degraded in an EspF-dependent manner, resulting in increased cleavage of caspase-9 and caspase-3, promoting apoptosis [52–55]. EspB can enter host cells autonomously and selectively induce the programmed cell death of monocytes [56]. In *Yersinia pestis*, the T3SS effector protein YopJ can promote apoptosis via the TLR4 signaling pathway [57]. YopP can cleave the caspase-8 substrate BID to promote apoptosis [58, 59]. SipB induces caspase-2 cleavage when *Salmonella* infects cells, resulting in caspase-1-independent apoptosis and cell death [60]. In addition, the T3SS effector proteins ExoS and ExoT in *P. aeruginosa* can also activate caspase-9 and caspase-3, leading to apoptosis [61].

**Figure 1:**
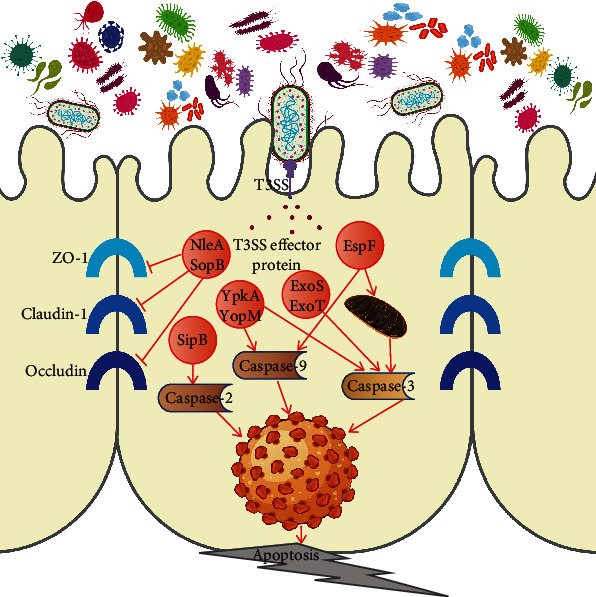
Effects of the T3SS on intestinal epithelial cells. The intestinal pathogenic flora destroys the tight junctions between intestinal epithelial cells via the effector proteins secreted. It also activates caspase-2, caspase-3, and caspase-9 in epithelial cells and macrophages to promote apoptosis.

Therefore, the T3SS may cause damage to the intestinal mechanical barrier, which can lead to the disturbance of the immune function of the intestinal epithelial cells. Therefore, the chemical barrier, which is composed of mucus and digestive juices secreted by intestinal epithelial cells and antibacterial substances secreted by normal bacteria, will also be destroyed (Figure 1). Dysfunction of the intestinal barrier increases its permeability to enteric pathogens and endotoxins, leading to systemic inflammation and aggravating the development of metabolic diseases [62–64].

## 4. The Function of Intestinal Pathogens' T3SS in Metabolic Diseases

Under normal physiological conditions, the intestinal barrier prevents bacteria and endotoxins from entering the blood and lymphatic circulatory system [65]. However, significant increases in intestinal permeability have been observed in patients with obesity, insulin resistance (IR), and associated cardiovascular complications [66]. The mechanism may be related to changes in intestinal pathogenic bacteria containing the T3SS, which increases intestinal barrier permeability and leads to several metabolic diseases.

### 4.1. T3SS and T2DM

T2DM has been a worldwide public health problem for many years and is associated with IR, insufficient insulin secretion, pancreatic islet cell destruction, etc. [67, 68]. It is mainly characterized by clinical metabolic syndrome based on insulin deficiency and hyperglycemia and mostly occurs in obese people [69].

Many studies have explored the presence of gut microbiota disturbances in patients with T2DM. In 2010, Larsen et al. analyzed the differences in the gut microbiota between T2DM patients and normal controls and found that the microbial community composition between the two groups was significantly different at the phylum level; the abundance of Firmicutes in the T2DM group was lower than that in the healthy control group, while the relative abundance of Proteobacteria and Bacteroidetes was higher in the T2DM group [70]. Later, Karlsson et al. found that the levels of opportunistic pathogens such as *E. coli* and *Bacteroidetes* in the intestinal tract of patients with T2DM increased, the proportion of Firmicutes decreased, and the content of probiotics such as Bifidobacterium significantly decreased [71]. Qin et al. found that the main genera in stool samples from patients with T2DM were opportunistic pathogens, such as *Bacteroides faecalis*, *E. coli*, *Clostridium symbiotica*, *Eggertella lentus*, and *Clostridium polymycota* [72]. Thus, the abundance of T3SS-containing bacteria such as *E. coli* was increased in the guts of T2DM patients. Although the relationship between the T3SS in *E. coli* and T2DM is not clear, it may be related to impaired intestinal barrier function and the consequent elevated levels of long-term chronic inflammation.

Opportunistic pathogens use the T3SS to increase intestinal permeability by destroying tight junction proteins such as occludin and ZO-1 [73, 74]. The damaged intestinal barrier continuously produces and physiologically transfers endogenous LPS to the intestinal capillaries via a TLR4-dependent mechanism, which upregulates the metabolic concentration of LPS in the plasma [75]. The metabolic concentration of plasma LPS triggers the metabolic disease of diabetes induced by a high-fat diet. When the metabolic concentration of LPS increases, the system composed of LPS and its receptor CD14 controls insulin sensitivity, sets the threshold for the occurrence of metabolic diseases, and ultimately aggravates the development of IR [76].

In addition, opportunistic pathogens aggravate the systemic inflammatory response and metabolic disorders via the NF-*κ*B pathway and MAPK signal transduction during infection [77–79]. For example, in EPEC and *Citrobacter* infection, the T3SS effector protein EspT induces the expression of proinflammatory cytokines COX-2, IL-8, and IL-1*β* by activating the NF-*κ*B, ERK1/2, and JNK signaling pathways in macrophages [80]. Similarly, after *Shigella* infection, the effector proteins IpgB2 and OspB can also be activated by mediating the NF-*κ*B, ERK1/2, and p38 signaling pathways to activate the release of neutrophil chemokines. The recruitment of neutrophils to the infected site can destabilize the epithelial barrier, thus promoting the invasion of *Shigella* into the colonic mucosa [81]. *Salmonella* uses many effector proteins, such as SopB, SipA, and SspH1, to regulate the NF-*κ*B signaling pathway. The effector protein SopB mediates the activation of the NF-*κ*B signaling pathway via phosphorylation and causes inflammation in host cells [82]. The effector protein SipA activates NF-*κ*B by activating nucleotides to bind the oligomeric domain 1 NOD1/NOD2 signaling pathway, which leads to an inflammatory response [83]. Innate immunity caused by *Salmonella typhimurium* infection requires SopE and SopE2 to activate Rho family GTPase and then the NF-*κ*B signaling pathway [84]. SopE activates Rac1 and Cdc42, triggering the NOD1 signaling pathway and the NF-*κ*B-dependent inflammatory response mediated by Rip2 [85].

**Figure 2:**
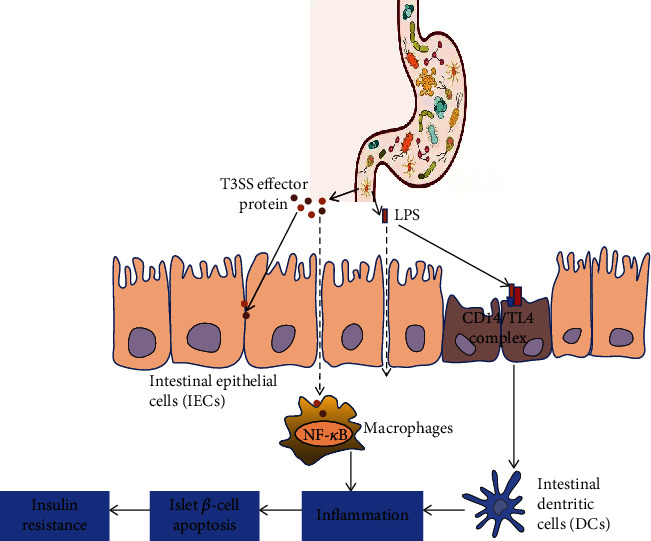
Effects of the T3SS on T2DM. The T3SS allows LPS to enter the host circulation by destroying the barrier function of the gut and leaking out to the internal environment, leading to an inflammatory status. Long-term chronic inflammation accelerates islet *β*-cell apoptosis and eventually leads to IR.

Long-term chronic inflammation disrupts the internal environment, affecting the balance of islet cells promoting apoptosis and antiapoptosis, promoting islet dysfunction, and accelerating islet *β*-cell apoptosis [86]. It has been reported that type 2 diabetic human islets *β*-cell have gradually deteriorated in function, diminished in mass, and show a higher level of apoptosis [87, 88]. As such, the damage and insufficient number of pancreatic *β*-cells induced by the T3SS leads to insufficient insulin secretion and IR and aggravates the development of T2DM (Figure 2).

### 4.2. T3SS and Gout

Gout is a metabolic disease with monosodium urate (MSU) crystal deposition caused by the disorder of purine metabolism and seriously threatens human health as a debilitating chronic inflammatory arthritis [89]. The pathogenesis of gouty arthritis is closely related to the body's production of several inflammatory factors such as IL-1*β*. In the inflammatory response mechanism, the NLRP3 and TLR signaling pathways cooperate to induce the maturation and secretion of IL-1*β* [90, 91]. The literature has reported that the activation of the NLRP3 inflammasome is a crucial signal transduction pathway for inducing the occurrence and development of gouty arthritis [92]. The basic structure of the NLRP3 inflammasome is a signal transduction complex composed of NLRP3, ASC, and caspase-1 [93]. Endogenous stimulatory factors such as MSU crystals can induce the body to produce a large amount of IL-1*β* and other inflammatory factors by activating the NLRP3 inflammasome signaling pathway, thereby inducing gouty arthritis [94].

Many studies have reported a significant correlation between gout and intestinal flora, and there are differences in the composition of intestinal flora in gout patients compared with normal people. Guo et al. analyzed the fecal bacteria of 35 gout patients and 33 healthy people and found that the diversity of intestinal flora in gout patients was significantly reduced. Moreover, their intestinal tracts were rich in *Bacteroides stercoris* and *B. xylanisolvens* and lacked Faecalibacterium pratense and Bifidobacterium pseudostrandum [95]. Another study performed 16S rRNA sequencing on stool samples from 58 primary gout patients and 53 normal controls and found increased levels of Proteobacteria and *Shigella* in the guts of gout patients [96]. Thus, the abundance of T3SS-containing bacteria such as *Shigella* was increased in the gut of Gout patients. Although the relationship between the *Shigella* T3SS and gout is unclear, it may be related to the induction of NLRP3 inflammasome signaling and increased inflammation.

*Shigella* deliver T3SS effectors into host cells to stimulate IL-1*β* secretion by the T3SS. *Shigella* delivers the T3SS effector protein IpaH7.8 to activate the NLRP3 and NLRC4 inflammasomes and induce macrophage pyroptosis [97]. Wang et al. found that IpaH4.5 interacts directly with NLRP3 to activate the NLRP3 inflammasome through increased K63-linked ubiquitination [98]. The activation of the NLRP3 inflammasome causes the body to produce an abundance of inflammatory factors such as IL-1*β*, thereby inducing gouty arthritis.

**Figure 3:**
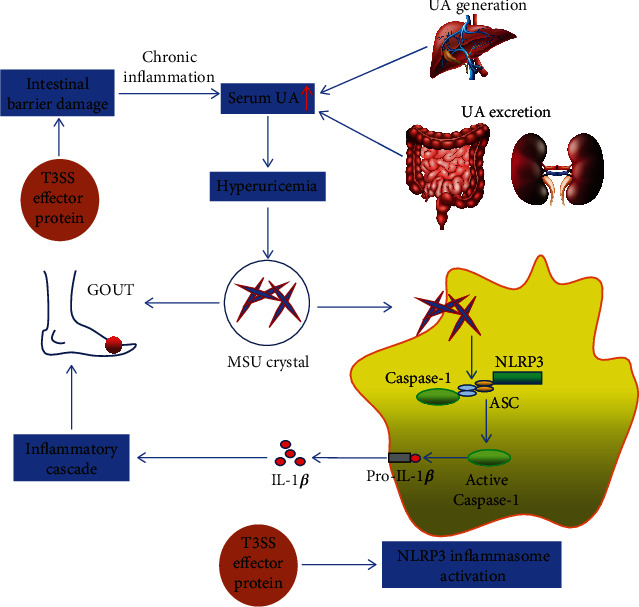
Effects of the T3SS on gout. The T3SS increases the secretion of LPS and inflammatory response. Intestinal barrier loss caused by the T3SS leads to intestinal-derived LPS translocation and chronic inflammation, increasing serum UA. Moreover, the T3SS may activate the NLRP3 inflammasome and lead to gout.

It has been reported that intestinal barrier dysfunction and increased intestinal permeability can lead to hyperuricemia in mice [99]. The T3SS in intestinal flora triggers and aggravates chronic intestinal inflammation, thereby destroying the intestinal mucosal barrier, resulting in increased intestinal permeability and the release of more inflammatory factors. This inhibits the expression of uric acid (UA) transporters in intestinal epithelial cells, hinders the excretion of UA in the intestine, and increases blood UA levels, thereby promoting the occurrence and development of gout. Long-term deposition of UA in the joints can also cause gout. As such, the T3SS may induce the production of endotoxins in the body, causing chronic inflammation and leading to the occurrence and development of gout (Figure 3).

### 4.3. T3SS and NAFLD

NAFLD is a chronic liver disease characterized by simple fatty liver (NAFL) or steatohepatitis (NASH), which eventually progresses to cirrhosis and liver cancer [100–102]. Growing experimental and clinical evidence suggests that the gut microbiota may be involved in the pathogenesis of NAFLD.

Patients with NAFLD have higher levels of Proteobacteria, Fusobacteria, and Actinobacteria than healthy individuals, while Prevotella and Bacteroidetes are less abundant [103, 104]. In addition to an imbalance in the Bacteroidetes/Firmicutes ratio and a decrease in bacterial diversity, some studies have found that the abundance of *E. coli*, *Lactobacillus*, and *Bacteroides* in the intestinal flora of NAFLD patients is significantly increased [105], while the abundance of *Enterococcus faecalis*, *Faecalibacterium prausnitzii*, and *Ruminococcus* is significantly reduced [106]. Thus, the abundance of T3SS-containing bacteria such as Proteobacteria is increased in the guts of NAFLD patients. Although the relationship between the T3SS in Proteobacteria and NAFLD is unclear, it may be related to intestinal permeability.

Patients with NAFLD exhibited increased intestinal permeability, which was positively correlated with the severity of fatty liver disease [107]. Under the same diet-induced conditions, claudin-deficient mice are more prone to NAFLD than wild-type mice [108]. Another study showed that an impaired intestinal barrier allows several intestinal substances, such as bacteria, endogenous ethanol, bacterial endotoxins, and bile acids, to enter the liver node through the portal vein to induce NAFLD [109]. These results suggest that intestinal barrier function is critical for the occurrence of NAFLD.

**Figure 4:**
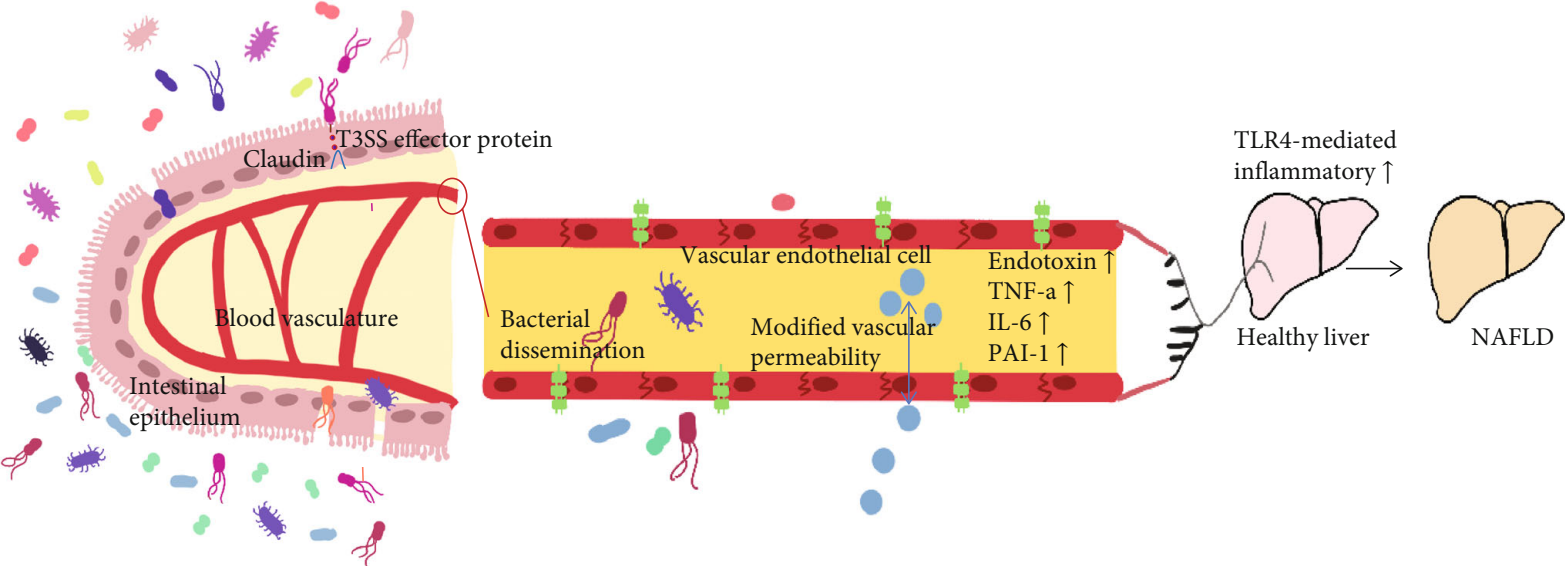
Effects of the T3SS on NAFLD. The T3SS in intestinal flora destroys the tight junction protein Claudin, resulting in higher serum endotoxin, TNF-a, IL-6, and PAI-1 levels. Macrophages and platelets emerge in the liver during TLR4-mediated inflammation and predispose the liver to NAFLD.

The intestinal epithelium's tight junction protein assembly (comprising ZO-1 and claudin) is altered due to the T3SS, resulting in a “leaky gut,” which translocates LPS into the bloodstream from the digestive tract lumen. LPS causes endotoxemia and the overproduction of proinflammatory cytokines, resulting in intestinal inflammation [110]. Endotoxemia can contribute to the progression of NAFLD in children with NAFLD who have higher serum endotoxin, TNF-a, IL-6, and PAI-1 levels [111]. As such, the T3SS may upregulate the TLR4 inflammatory pathway in the liver, leading to the secretion of inflammatory factors, inducing the activation of macrophages and platelets, and thereby promoting the development of NAFLD (Figure 4).

## 5. Measures Against the T3SS in Intestinal Pathogens

The T3SS genes encoded by pathogenic bacteria are often the source of pathogenicity and virulence, and their possible relationship with metabolic diseases has been speculated. Therefore, can we prevent the occurrence and development of metabolic diseases by regulating the expression of the T3SS genes in pathogenic bacteria?

### 5.1. Improving the T3SS With Diet

The gut microbiota maintains a relatively steady state in healthy humans. However, once the host environment changes, such as changes in dietary habits or antibiotic use, the commensal bacteria in the gut also change, increasing the likelihood of infection. Some pathogenic bacteria use this opportunity to disturb the bacterial flora, ingest previously inaccessible nutrients, and then proliferate in the gut. For example, succinate levels are significantly elevated when dietary habits are altered or when intestinal inflammatory reactions such as antibiotic-associated diarrhea occur [112]. A recent study examined changes in EHEC and *Citrobacter* gene expression when succinate was elevated and found that the expression of one-fifth of the genes, including the T3SS genes, was activated [113]. This phenomenon indicates that the expression of the T3SS genes is affected by human dietary habits.

Succinic acid can promote the expression of the LEE genes, including the T3SS genes, in EHEC and *Citrobacter*. In addition to succinic acid, A/C pathogenic bacteria such as EHEC can use ethanolamine, fucose, and galacturonic acid, as signals to regulate the expression of LEE genes [114–116]. Therefore, we should consume less foods rich in these ingredients and more foods rich in dietary fiber.

Maintaining good eating habits is very important for a person's health. Extensive literature supports the role of dietary fiber in health, including a dose–response relationship between higher fiber consumption and lower mortality [117]. Marques et al. found that a high fiber intake increased the abundance of acetogenic bacteria and altered the gut microbiota. Fiber and acetate both reduced gut dysbiosis, measured as Firmicutes-to-Bacteroidetes ratio, and increased the prevalence of Bacteroides acid bacteria [118]. Dietary fiber contains microbiota-accessible carbohydrates (MACs) that support gut microbiota diversity and metabolism as well as short-chain fatty acids (SCFAs) that benefit gut health.

In the intestinal mucosa, SCFAs, which are produced by the fermentation of dietary fiber by the gut microbiota, play an important role in the absorption of nutrients, facilitate colon cell proliferation, maintain epithelial tight junction integrity, and reduce intestinal barrier permeability [119, 120]. Recent studies have shown that SCFAs inhibit epithelial cell apoptosis and prevent intestinal bacterial toxin translocation [121]. SCFAs can also regulate the expression of bacterial toxin receptors and VFs, including T3SS and epithelial adhesion factors of certain intestinal pathogens [122, 123]. For example, butyrate and propionate in SCFAs can affect the pathogenicity of *Salmonella* in vivo and limit its invasion into host cells [124, 125].

Li et al. used a fecal metagenomic dataset to compare the characteristics of and changes in VF genes in gut microbes before and after high-fiber dietary intervention. In both cohorts, a high-fiber diet reduced the abundance of VFs, and pathogen-specific genes, including those related to invasion and the T3SS, were suppressed [126]. Wastyk et al. also observed cohort-wide reductions in many markers of inflammation in individuals consuming high-fiber foods, along with increased microbiota diversity [127]. Therefore, a high-fiber diet can inhibit the expression of the T3SS and improve the occurrence and development of metabolic diseases.

### 5.2. Improving the T3SS With T3SS Inhibitors

As antibiotic resistance has grown and drug development pipelines have become ineffective, treating bacterial infections has become increasingly difficult. Antibiotics target several processes to ensure bacterial growth and survival, such as DNA replication, protein synthesis, and the production of cell walls. Consequently, antibiotics exert high selective pressure on resistance mutations, and antibiotic resistance usually occurs only after a few years of clinical use [128]. “Virulence blockers” have emerged as a promising alternative to combat infection [129]. Due to targeting VFs, these drugs disarm the pathogen, allowing the host's immune system to eliminate it. As virulence mechanisms are generally not required for bacteria to grow, selective pressure-driven resistance should be minimal. The T3SS genes in *Yersinia pestis* were mutated in a study, and the strain became nontoxic when injected directly into the bloodstreams of susceptible mice [130]. Therefore, the T3SS is essential for complete virulence. Considering the increasing levels of antibiotic resistance in T3SS-containing pathogens [131], alternative treatments must be found. A high level of conservation has been found for the T3SS, it plays a key role in virulence, and it is not found in nonpathogenic bacteria [132], making it a great target for antibacterial therapy.

Over the past two decades, several promising inhibitors of the T3SS have been discovered using high-throughput screening (HTS) from small-molecule libraries [133], as well as rational design and screening of natural sources [134–137]. In 2002, the first T3SS inhibitors were identified by Linington et al., and they had no effect on bacterial growth [138]. Kauppi et al. identified a variety of chemical classes that inhibit the T3SS [139]. Nordfelth et al. demonstrated that mammalian cells were protected against T3SS-mediated cytotoxicity by these inhibitors [140]. According to Hudson et al., T3SS inhibitors inhibit both the *Yersinia pseudotuberculosis* T3SS and the *Salmonella* SPI-1-encoded T3SS, resulting in minimized virulence phenotypes in animal experiments, suggesting broad–spectrum T3SS inhibitors are desirable [141]. Using a T3SS inhibitor for *Chlamydia trachomatis* indicates that it seems to be an effective topical prophylactic against this sexually transmitted pathogen [142]. In clinical trials, engineered antibodies that block the T3SS of *Pseudomonas aeruginosa* have shown promise for multiple indications [143].

Recently, whole cell-based HTS for the identification of T3SS inhibitors has shown that small molecule compounds such as salicylic hydrazide, salicylanilide, sulfonamidobenzanilide, benzimidazole, thiazolidinone, and some natural products are effective against many T3SS-utilizing pathogens, including *Yersinia*, *Chlamydia*, *Salmonella*, large-intestine Bacillus, *Shigella*, and *Pseudomonas* [144–148]. In cell culture models, many T3SS inhibitors have shown promise; however, further studies in animal models are needed. These T3SS-specific antibacterial approaches may be effective therapeutic options for many clinically relevant Gram-negative pathogens. To gain clinical approval, however, these inhibitors must be more effective than antibiotics in treating bacterial infections [149] (Table 2).

## 6. Conclusions and Future Perspectives

Metabolic diseases have become a worldwide health problem. Long-term intake of high-fat and high-sugar diets can change the intestinal environment, especially the composition of intestinal microorganisms that weaken intestinal barrier function and increase its permeability [150], cause an increase in endotoxin levels, induce chronic low-level inflammation, and ultimately lead to metabolic diseases. In the past few decades, many researchers have used metagenomic sequencing technology to compare the intestinal microbiomes of different groups and have confirmed that changes in the intestinal microbiota significantly impact metabolic diseases such as T2DM and NAFLD [151].

The gut microbiota has significant therapeutic potential and an important role in metabolic diseases. However, most existing studies are cross-sectional or only demonstrate microbial modulation and specific disease associations. To determine a causal link between the intestinal microbiota and metabolic diseases, further prospective studies and confirmation of the underlying molecular mechanisms are needed. Since some VFs produced by the gut microbiota play a role in triggering host inflammation, we focused on the T3SS, a typical class of VFs.

It is crucial to acknowledge that the T3SS is not universally present among commensal bacteria, and its specific functions and effects are likely modulated by a range of factors. Notably, there exists a competitive dynamic between commensal bacteria and enteric pathogens. Enteric pathogens surmount this competitive barrier through the utilization of the T3SS, which facilitates the injection of a repertoire of virulent proteins into host colon cells, thereby potentially inducing symptoms of inflammation and infection [33, 34, 45].

The prevalence of the T3SS is contingent upon the specific commensal bacterial species and their respective environmental contexts. For instance, within the gastrointestinal tract, certain commensal strains of *E. coli* may harbor the T3SS. While the majority of *E. coli* strains function as commensal organisms, particular strains such as EPEC and EHEC possess the T3SS [31]. These pathogenic strains utilize the system to secrete VFs into host cells, thereby eliciting an immune response.

This review began with an introduction to the role and function of the T3SS and analyzed the latest studies reporting changes in the intestinal microbiota of people with different diseases, screening out those reporting T3SS-containing flora with significant changes. Based on the role of the T3SS and the pathogenesis of metabolic diseases, we can deduce their potential relationship.

The T3SS can cause intestinal physical barrier damage, leading to dysfunction of intestinal mucosal epithelial immune cells, and it allows LPS to enter the host circulation and bind to Toll-like receptors and CD14 on the surfaces of immune cells to form a complex, which may activate immune cells and ultimately cause chronic systemic inflammation. Chronic systemic inflammation is an important factor in the formation of metabolic diseases.

The abundance of T3SS–containing pathogenic bacteria in the gut microbiota is so small that there are few studies on the relationship between T3SS and metabolic diseases. Nevertheless, it is a worthwhile new research direction because it focuses on the VFs of pathogenic bacteria rather than the composition of the intestinal flora. In the future, the specific mechanism of the effect of the T3SS on metabolic diseases needs to be further explored, whether it is to study the mechanisms of metabolic diseases or to develop drugs for metabolic diseases with potential targets.

## Figures and Tables

**Table 1:** Core structural components of the T3SS in intestinal pathogens [33–37].

**Core structural components**	**EPEC and EHEC**	***Yersinia* spp.**	***Salmonella* spp.**	***Shigella* spp.**
Basal body	EscC, EscD, EscJ, EscF, EscI, and EtgA	YscC, YscD, YscJ, YscF, and YscI	InvG, PrgH, PrgK, PrgI, PrgJ, and IagB	MxiD, MxiG, MxiJ, and MxiH
Export apparatus	EscU, EscV, EscR, and EscS	YscU, YscV, YscR, and YscS	SpaS, InvA, SpaP, and SpaQ	Spa40, MxiA, Spa24, and Spa9
Cytoplasmic ring	SepQ	YscQ	SpaO	Spa33
ATPase complex	EscN, EscL, EscO, and EscK	YscN, YscL, YscO, and Ysck	InvC, OrgB, InvI, and OrgA	Spa47, MxiN, Spa13, and MxiK
Regulators	EscP, SepL, and SepD	YscP and YopN-TyeA	InvJ and InvE	Spa32 and MxiC
Translocators	EspB, EspD, and EspA	YopD, YopB, and LcrV	SipC, SipB, and SipD	IpaC, IpaB, and IpaD

**Table 2:** Measures against the T3SS in intestinal pathogens.

**Type**	**Component**	**Function**	**References**
Harmful foods	Succinic acid	Promote the expression of the T3SS.	[112–116]
	Fucose		
	Ethanolamine		
	Galacturonic acid		

Dietary fiber	Coarse grain	Contain short-chain fatty acids that regulate the expression of the T3SS genes.	[118–125]
	Vegetable		
	Fruit		
	Beans		

Inhibitors of the T3SS	Salicylic hydrazide	Have shown promise, but further studies in animal models are needed.	[134–143]
	Sulfonamidobenzanilide		
	Salicylanilide		
	Benzimidazole		
	Thiazolidinone		

## Data Availability

Data availability is not applicable to this article as no new data were created or analyzed in this study.
